# Patient and Staff Insights on Digital Care Pathways for Patients With Low Back Pain in the Emergency Department: A Qualitative Study

**DOI:** 10.1111/hex.14182

**Published:** 2024-08-16

**Authors:** Emily C. Bell, Hazel Heng, Nicole Alousis, Matthew G. King, Andrew Hahne, Thomas Collins, Katharine See, Tracey Webster, Elisha O'Dowd, Paul Jackson, Adam I. Semciw

**Affiliations:** ^1^ Discipline of Physiotherapy La Trobe University Bundoora Australia; ^2^ Department of Allied Health Northern Health Epping Australia; ^3^ Digital Health Division, Clinical Leadership, Effectiveness & Outcomes Northern Health Epping Australia

**Keywords:** back pain, digital care pathway, digital health, physiotherapy, qualitative, rehabilitation

## Abstract

**Background:**

Back pain is a huge global problem. For some people, the pain is so severe that they feel the need to present to an emergency department (ED). Our aim was to explore patient and staff perspectives for the development of a digital care pathway (DCP) for people with back pain who have presented to ED, including acceptability, barriers and facilitators.

**Methods:**

We used a descriptive phenomenology approach using semi‐structured interviews with patient and staff participants at a tertiary hospital. Interviews were transcribed and data codes were developed using inductive thematic analysis. Themes were discussed between researchers until consensus was achieved.

**Results:**

A total of 16 interviews were carried out, half of which involved patient participants. We identified three major themes: (i) expectations and experiences of staff and patients with low back pain in ED; (ii) a digital care pathway can empower patients and support clinicians in providing care; and (iii) acceptability, barriers, facilitators and recommendations of engaging with a DCP to track the trajectory of back pain. Each theme was further categorised into subthemes.

**Conclusion:**

Introducing a DCP was perceived as acceptable and beneficial by patients and staff. Both groups were aware of the potential participant burden if surveys were too long. Introducing a DCP could be a valuable adjunct to current management care models, providing a standardised source of education with the potential for individualised tracking and monitoring. The design and development of a DCP will need to consider reported facilitators and address perceived barriers for engagement.

**Patient or Public Contribution:**

This project sought insights from patients and staff about a digital care pathway. This forms the first step of patient and consumer consultation before implementing a digital care pathway. All consumers were offered the opportunity to review their responses and our interpretation.

## 1 Introduction

### 1.1 Background

Back pain affects one in six people in Australia, with higher prevalence rates observed in older individuals and those from lower socioeconomic areas [1]. It is the leading cause of burden for people aged 35–54 years [1, 2] and is the most common reason for work absenteeism and premature departure from the workforce [3, 4, 5]. Given this high prevalence, it is not surprising that back pain is a common presentation in Emergency Departments (ED). A meta‐analysis estimates presentation rates of 4.39% (95% confidence interval [95% CI]: 3.67, 5.18) [6]. Admission rates are varied across the globe, with Australian data indicating particularly high rates (17%–53%) [7]. This is concerning, given that back pain presentations are the 5th most common cause of ED presentations in Australia [8]. For these patients, pain is severe (average 2 out of 10 points worse than back pain in general practice [9]) and impacts their quality of life [10] and their ability to attend work or engage in the community [11]. For patients who are subsequently admitted, the cost may reach an average of $15,000 AUD [12]. Australian EDs aim to treat and discharge patients from ED within 4 h. In 2022–2023, Australian EDs were completing just 56% of visits within 4 h (down from 61% in 2021–2022) [8]. With annual growth in presentations in one Australian state estimated to be around 3.2% [13], and representation rates ranging from 5.6% to 11% [13], action is needed to keep non‐emergent back pain cases from presenting to ED.

### 1.2 Importance

A digital care pathway (DCP) is a novel intervention that may enhance management for patients who present to ED with back pain. DCPs seek to integrate digital technologies into clinical care, to facilitate efficient patient workflows, monitor patient progress or deterioration and/or enhance synchronous or asynchronous communication between the patient and their healthcare team [14]. DCPs have been used for chronic low back pain in the community in the United States, with patients who completed the programme reporting improved pain (52%–64%) and function (31%–55%) [15]. However, the success of these programmes is based on adherence, uptake and motivation by patients and clinicians involved. For example, only 61% of people with chronic low back pain randomised to a DCP actually completed the 12‐week programme [15]. This is supported by a feasibility study in which only 30% of patients undergoing spinal surgery completed at least one postoperative outcome measure, indicating poor engagement with the DCP [16]. There is a mismatch in the high uptake of digital technology since the COVID‐19 pandemic and the digital literacy of clinicians delivering these programmes [17]. There can also be a reluctance of health services or clinical staff to implement and deliver digital programmes, given that public health services are often funded by patient volume, whereas DCPs aim to facilitate care at home [18]. This may require challenging creative accounting practices in the development of business models to support DCP implementation [18]. There is a clear need to understand the specific challenges and facilitators of DCP uptake from a patient and staff perspective before implementing these models.

### 1.3 Goals of This Investigation

The aim of this study was to collate patient and staff perspectives for the development of a DCP for people who present to ED with back pain. This includes the frequency, duration and format of the DCP, desired education content and barriers and facilitators.

## 2 Methods

### 2.1 Study Design and Setting

This study constitutes phase 1 of an ongoing study to design, implement and evaluate a DCP for people with back pain presenting to ED [19]. We conducted a qualitative study using a descriptive phenomenology approach. Semi‐structured interviews explored the perspectives of patients with back pain and staff who treat them about using a DCP. Interviews were led by an experienced female physiotherapist and researcher (E.C.B.). Reporting of this study was guided by the consolidated criteria for reporting qualitative research (COREQ) [20] checklist. This project was approved by the St Vincent's Hospital Human Research Ethics (2022/PID06476), and governance approval was received from La Trobe University (HEC#206/22) and Northern Health (SSA/89217/NH‐2023).

The research was conducted at Northern Health in Victoria, Australia, where eligible individuals suffering from back pain were recruited from the ED. Northern Health serves as a primary provider of acute, maternity, subacute and specialist healthcare services in the rapidly expanding outer north region of Melbourne. The ED handles over 120,000 visits annually.

### 2.2 Selection of Participants

#### 2.2.1 Recruitment

Patient volunteers gave verbal consent after their ED attendance to be contacted by a member of the research team via phone. Following the phone call, volunteers were sent the participant information statement via email. Staff volunteers were recruited via advertisements and email. Volunteers who gave digital consent via Research Electronic Data Capture (REDCap), a secure online data centre hosted by Northern Health, were included. Participants needed to be able to understand written and verbal English.

#### 2.2.2 Eligibility

##### 2.2.2.1 Patients

People who presented to the Northern Health ED with musculoskeletal back pain and were not subsequently admitted to a ward (e.g., were referred to the back pain outpatient clinic) were eligible to be recruited as per our protocol [19]. Back pain was diagnosed via clinical assessment by emergency clinicians, including physicians, physiotherapists and/or clinical nurse specialists.

##### 2.2.2.2 Staff

Staff members were eligible to be included if they were patient‐facing health professionals who treated people with back pain at Northern Health ED.

### 2.3 Data Collection

Semi‐structured interviews, guided by a topic guide for patients and one for staff (Appendix S1), were developed using the Consolidated Framework for Implementation Research (CFIR) [21]. The questions were also informed based on the clinical and research experience of physiotherapists (E.C.B., M.G.K., and A.I.S.). Questions were not piloted. Questions were based around participants' experience with back pain and management, acceptability, barriers and facilitators for engaging with a DCP. Three staff participants were known to the interviewer briefly in a limited professional capacity before the interviews. The remaining participants were not known to the interviewer and were introduced to the researcher and project aims at the beginning of their interview. Interviews were conducted online via Zoom (San Jose, CA) between September and December 2023. They were audio‐recorded and transcribed using Otter.ai (Mountain View, CA). Participants were provided with their transcript within 2 weeks and allowed to provide feedback within the next 2 weeks. Transcripts were deidentified and participants were assigned a code (P for patient; S for staff). No repeat interviews were conducted; field notes were collected to add depth to the interview findings. Data analysis was conducted immediately to determine whether new information was being collected. We collected participant sex, age (patient participants only), years treating back pain (staff participants only) and average length of interviews. Three staff participants are co‐authors (N.A., T.C., and P.J.) to provide robust suggestions to improve the clinical applicability of our findings.

### 2.4 Data Analysis

Inductive reflexive thematic analysis [22] was supported by NVivo software (QSR International Pty Ltd, Melbourne, Australia). Analysis was conducted by two researchers with previous qualitative and mixed‐methods experience in musculoskeletal conditions (E.C.B. and H.H.) [23, 24, 25, 26, 27]. Researchers first closely reviewed each transcript to obtain an overall picture of the data. Researchers discussed potential codes, and then assigned codes to each key issue to establish a thematic framework. Through discussion, codes were grouped to form subthemes and subthemes were grouped to form overarching themes. Themes were not predetermined, and all transcripts were included in the analysis to contribute to the thematic framework. Identified themes were discussed and refined between researchers at six meetings until consensus was achieved. Once researchers determined that data saturation had reached, with no new themes being identified from the data, interviews were ceased. Demographic details were reported descriptively with mean and standard deviation (SD).

## 3 Results

### 3.1 Characteristics of Study Subjects

We recruited 16 participants, including eight patients and eight staff. A further five patients were eligible but declined to participate, citing lack of interest (*n* = 5). Seven (44%) participants were female. Patient participants had an average age of 48 years (SD: 12). The mean length of the interviews was 43 min (SD: 7 min). Participant demographics are shown in Table 1. Transcript feedback was provided by zero patients and three staff participants, following further time to reflect on the interview questions. Feedback included alterations to quotes and information they wanted to add.

**Table 1:** Demographics.

	Patients (*n* = 8)	Staff (*n* = 8)
Sex, *n*% female	3 (38%)	4 (50%)
Age, mean (SD), years	48 (12)	N/A
Average length of interviews, mean (SD), min	40 (6)	45 (7)
Years of experience, years (SD)	N/A	16.13 (5.28)

Abbreviations: *n*, number of participants; N/A, not applicable; SD, standard deviation.

### 3.2 Main Results

**Figure 1:**
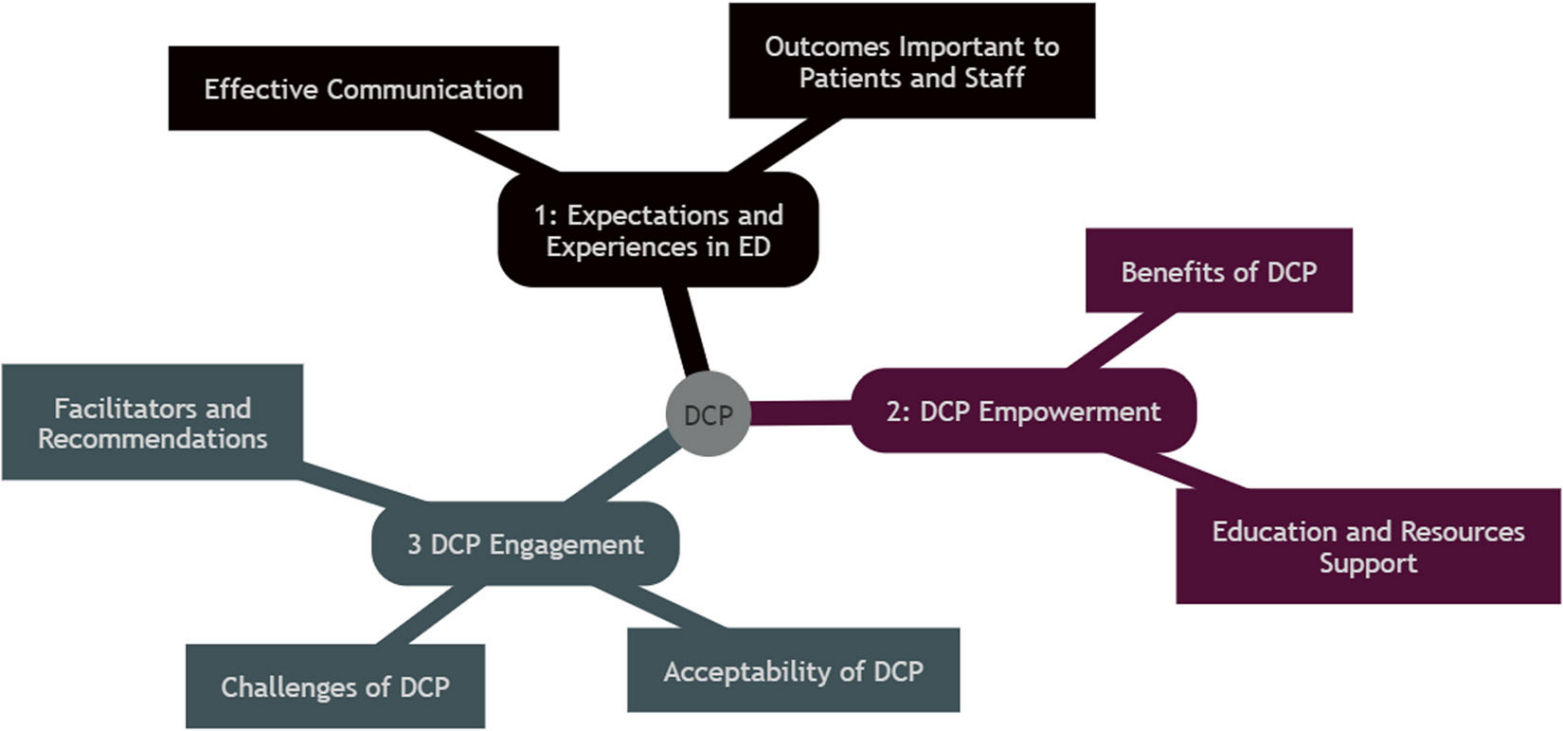
Mind map illustrating the themes and subthemes.

Three major themes were identified from the data, including (Figure 1) (1) expectations and experiences of staff and patients with low back pain in ED; (2) a digital care pathway can empower patients and support clinicians in providing care; and (3) acceptability, barriers, facilitators and recommendations of engaging with a DCP to track the trajectory of back pain. Each theme was further categorised into subthemes. For the first theme, two subthemes related to how people who presented to ED with severe low back pain preferred effective communication to manage expectations around their stay in ED and that there were several outcomes that were important to patients and staff in ED. The second theme had two subthemes: associated with participants predicting the benefits of a DCP and how a DCP could empower and support patients through education and resources. The final theme was categorised into three subthemes: (i) the perceived acceptability of the DCP, (ii) perceived challenges around implementing and engaging with the DCP, and (iii) facilitators and recommendations for improving engagement with the DCP. Full details of each theme, subtheme and supporting quotes can be found in Appendix S2.

## 4 Theme 1 Expectations and Experiences of Staff and Patients With Low Back Pain in ED

### 4.1 People With Low Back Pain Who Present to ED With Severe Symptoms Want Effective Communication to Set Expectations Around Their ED Journey

Patient participants identified acute back pain episodes as a severe debilitating condition, leading them to seek urgent care in ED. By doing so, some patients reported experiencing feelings of guilt and anxiety as they were unsure if their condition was serious enough to warrant emergency care (Box 1). Patient participants wanted clear expectations about their journey through ED, which could be achieved through effective communication and education while in the hospital. This was reflected by two patient participants who reported having a positive experience as they felt that they had sufficient information and care (Box 1). In contrast, two patients attributed negative ED experiences to insufficient communication (Box 1).

Box 1 **Communication is important for patients in ED with low back pain to manage expectations.**

“… the Friday when I went to the emergency room, I just had the pain all down my left side and down my left leg* to my foot to the point where I actually couldn't even walk, … the pain was just so excruciating. I couldn't even function” *P4*

“[I felt] guilty coming into the hospital with just a back pain and not life‐threatening illness. But I was just in so much agony. I kept saying I'm so sorry I'm here.” *P6*

“[I] didn't even know that [I] will have this follow up. You know, I will be taken care of by a physio … [at the back pain] Hot clinic. … I went to the clinic but you don't have any information from the beginning. I thought it would be less like this, you know?” *P2*

“… everything that was explained to me at the hospital I thought was fine. I didn't feel out of the loop or anything.” *P5*

“… You'll be given painkillers. You know, someone will make an assessment on you. Am I having a scan tonight? Am I staying overnight? Just to also make it easier for my wife to sort of plan around” *P7*

“I tried at least walking around as much as possible. But … what if I'm at home … I'm just sitting there constantly taking painkillers. … is that going to help me in the long run? … in the hospital if I was told, like, just by sitting in the chair, doing the pelvic tilt or laying in bed, do the pelvic tilt. … maybe I wouldn't have been in there as long and the pain would have subsided a lot quicker” *P3*

*Referred leg pain is a common symptom for people with back pain [28].

### 4.2 Outcomes That Are Important to Patients and Staff

More than half of the participants agreed that outcomes that were important to them included pain management, quality of life and mobility. However, staff participants reported additional outcomes that were important to consider, such as the patient's ability to be discharged home, empowering patients through education and identifying serious pathology for further examination (Box 2). Furthermore, staff acknowledged competing interests and challenges when delivering evidence‐based holistic care. These include organisational pressures to discharge or transfer patients from ED, time constraints and managing patient expectations. One staff participant reported that this may result in staff making decisions that do not align with current evidence such as unnecessary imaging (Appendix S2).

Box 2 **Patients and staff place importance on various outcomes.**

“I want to achieve a full recovery. … that I can conduct any physical activities … without fearing or thinking in the back of my mind, if I bend over here is my back going to go out again, or … that if it does happen again, it won't be as painful as it is now. … I don't want to be restricted in certain activities. … I don't want to stop playing golf for instance or doing something active like kicking the footy or playing cricket.” *P7*

“… to be able to live without pain. … just to be free to move … don't have to think twice before doing anything … Yeah, better life.” *P2*

“If I'm being patient focused, it's obviously to provide reassurance and education and adequate pain relief, and a plan post to discharge for the patient so that they leave comfortable, that they know that they that their severe pain is not life threatening and that they know what to do. And who to go to.” *S6*

“I think education for patients, it's pain relief. … to communicate the fact that they're not going to [sic] damage their back by doing things. … If you pick up something from the floor … it might cause pain. … there's a difference between pain and damage.” *S3*

“So from an analgesic point of view, enough to mobilise and be able to ideally return home under the same day to avoid keeping them in hospital and some of the things that can happen as a result of staying in hospital too long. Secondary kind of illness, lots of imaging, lots of Bloods, lots of communication. All of those types of things are avoidable if we can get people comfortable. And yeah, and get them home.” *S2*

“… making sure we're not missing serious pathology, and then patient expectations. And then kind of assessing level of functional ability, so mostly aiming to get people home. I don't know that keeping them in our hospital really helps a lot of them.” *S4*

“My role in the emergency department is to do those things for the patient but also in a very timely manner, and to discharge wherever possible and not admit wherever possible.” *S6*

“frankly speaking, the most important outcome for me is whether I can get them home and out at the emergency departments in a timely fashion.” *S7*

## 5 Theme 2: A DCP Can Empower Patients and Support Clinicians in Providing Care

### 5.1 Participants Anticipate Benefits Being Associated With the Introduction of a DCP for People With Back Pain

Participants felt that introducing a DCP for people with back pain could be beneficial in various ways. Both patient and staff participants agreed that remote monitoring via the digital care pathway may reduce physical health visits (Box 3). Patient participants viewed tracking personal progress and benchmarking against aggregate data as valuable for managing their symptoms and expectations. They also felt that having a health professional monitor their progress through the DCP would be helpful. Staff participants viewed the DCP as a method to manage patient expectations around back pain and provide predictive data to clinicians around ED re‐presentations and chronic pain.

Box 3 **Participants expect several benefits from implementing and engaging with a DCP.**

“Because for me, it's also would be nice to know that this is actually normal, even though the doctors had said so it would also be nice if there was some data showing. This is normal and I'll get the feeling back in my foot at some point.” *P4*

“… Yeah, [graphs] would be good because then I can say, oh, okay, the pain decreased because I did this extra today.” *P3*

“Yes, predictions. Yeah, definitely will be good to have for [returning to] work also. Yes, it will be helpful, it's my responsibility also to be able to be back to work as soon as possible. And to get because I have to answer to, to my boss, my bosses also. So yeah, predictions will be very important.” *P2*

“Yeah, but it would be nice. To have you know, a trained person actually monitoring and, and knowing that there is improvements, and they can see the improvements.” *P4*

“… I suppose if they're monitoring my wellbeing and if they feel like perhaps I'm lagging in a certain part of my recovery, they could notify me I suppose, or, you know, if they feel like I'm not exercising as often as I should, to help with the treatment of my injury. That'd be a nice prompt, I suppose. That'll be beneficial.” *P7*

“there's more points of contact between the healthcare provider and the patients. I think it makes them feel more validated, maybe makes you feel less lonely. I think it may give them a sense of comfort and justice with pain isn't just the pain itself.” *S3*

“to ease the pressure on GPs and, and hospital you know, emergency hospitals and all that things, you know, if I could manage, you know, the pain. I wouldn't call the emergency department and go there.” *P8*

“I wonder if it would be useful for clinicians involved, like I think, if you could give data to patients saying, you know, we've collected data of 30 patients and you know, 75% of them are better or feeling better at 6 weeks. I think that's positive hope for someone. Everyone assumes they're going to be in that 75%. I love that data. I think it's really powerful for patients to say, you know, like, I think in shared decision making and explaining illness trajectory, I think it's really helpful.” *S4*

“The thing that's tricky, from our point of view is we do really want to be collecting routine data that we can kind of compare these patients against and also stratify, in particular, their risk of re‐presentation and chronicity of complaint.” *S2*

### 5.2 A DCP Could Improve Patient Care by Empowering and Supporting Patients Through Education and Resources

Most participants agreed that reassurance and education about returning to usual activities would help patients manage their back pain. Some staff participants were aware that patients may lack an understanding around the imaging indications, thus feeling that managing patient expectations through the DCP was key. This was also reflected in two patient participant interviews where they recognised their limited knowledge around imaging (Box 4). Staff participants also felt that managing patient expectations around surgery was important in cases where surgery is not indicated in the first instance. Both staff and patient participants indicated that education around common pharmacology for back pain would be helpful in empowering patients to manage their analgesia independently. Two participants indicated an interest in resources linked to psychological services (Box 4). One staff member and one patient reported contrasting views on the importance of dietary advice (Appendix S2).

Box 4 **Providing education and resources through a DCP could empower patients and enhance care.**

“we want to get across in reassuring patients that it is safe to move. Highlighting to them that some gentle exercises and progressive exercise as appropriate can help and that that's a treatment.” *S1*

“I think reassurance is a big thing that, yeah, you're not alone. And you're not, you know, crazy or overreacting for going to the hospital in the first place. I think it probably would have made me feel a little bit more at ease knowing that it actually is such a big problem and one in five or one in six suffer from it and present to the hospital …” *P4*

“there was that assurance and that little bit of education as well in how to live with this back pain …” *P6*

“Highlighting that even if scans are not required at the moment that that's not something that's permanent, and if the situation gets worse than that can be revisited and empowering them to understand what are concerning signs and symptoms that justify coming to an emergency department again …” *S1*

“normalising the fact that a lot of people don't actually need scans, because isn't going to change our management …” *S5*

“Yeah, maybe how much they're [imaging] actually needed? Because I had, first I had an ultrasound and I had an X‐ray. Then I had a CT scan. But when I saw the [physio] at the back clinic, she said that none of them were really necessary in the beginning.” *P4*

“And the analgesic information that we give people we throw a whole range of new medications to people on a day that they're in crisis and often drugged and then wave them goodbye. And expect them to remember that they should take two paracetamol every four to six hours, no dramas whether they've taken food or not, but the ibuprofen is four to six hourly, also with food. And then on top of that, they can take one Endone tablet.” *S2*

“multimodal care and like you know, kind of simple analgesia … I think that's useful.” *S4*

“a video addressing why surgery is not the first choice.” *S5*

“a lot of people get referred for surgery that don't need it for back pain.” *S1*

“it's all about modifying expectations, so that people and I mean, it's not just the patients because I get people sent in from GP's who are coming in saying ‘the GP said you would do an MRI and the GP said, I probably need surgery’” *S7*

“I'd be pretty interested in if you guys were going to include some like recommendations for mindfulness or meditation apps, like I think there's a lot of room for people to use them for pain modulation … So I wonder if that might be like a little section about with psychological support and ways to focus on other things.” *S4*

Abbreviation: endone, brand name for oxycodone.

## 6 Theme 3 Acceptability, Barriers, Facilitators and Recommendations of Engaging with a DCP to Track the Trajectory of Back Pain

### 6.1 Perceived Acceptability of DCP Delivery Mode, Frequency, Duration and Security

Participants had similar views around the perceived acceptability of the DCP. All participants preferred electronic delivery of surveys; however, there were differing opinions on whether emails or texts were more appropriate. Patient participants identified an acceptable frequency of surveys to be fortnightly and that the ideal survey duration should be under 10 min. One staff participant agreed that surveys should be short in duration to minimise patient burden (Box 5). A patient participant expressed that completing a survey during the acute episode would be a challenge (Box 5). The same participant felt that completing the survey shortly after the pain episode would result in more accurate recall than completing the survey weeks later (Appendix S2). Regarding data security, although some patient participants had concerns, most were realistic about the increased prevalence of hacking and were not specifically concerned about their health data (Box 5).

Box 5. **Perceived acceptability of DCP delivery mode, frequency, duration and security.**

“electronic I think. … if it's older generations, it might be slightly more challenging, but I think if we've got multi, long linguistic and culturally diverse access, I think most people are used to doing forms online, think it's quick and most people almost everybody has a phone these days. … that's quicker from a patient satisfaction point of view. I assume also much easier from a data collection point of view. … digital is where we should be going for everything. Also, climate point of view” *S4*

“Or through email is easy. We can, you know, communicate … easily …” *P1*

“at the time that I was in the waiting room of the hospital, if I was trying to fill out forms and watch videos, … I was in so much pain. I wasn't concentrating fully so it might not have been sort of taken in as well as when I was feeling better.” *P4*

“I wouldn't mind spending at least maybe 10 min in the morning and maybe 10 min in the evening [on activities and education] … On a weekly basis, I can see the gradual improvements.” *P7*

“Very good idea within 2 weeks, that should be improved” *P8*

“it needs to be a simplified or time limited survey that doesn't impede on engagement … ” *S5*

“It's going to healthcare professionals, it's not going to anything else. So I don't think I need to worry about that” *P6*

“lately we've been hearing about this hacking. In all things that's scary to me, you know, it's really scary. You feel sometimes unsafe. And, I mean, it's a lot of things, that may happen, or they may have an access or somewhere, especially an insurance or whatever … just [sharing the information] between my GP and my specialist, that should be enough.” *P8*

### 6.2 Perceived Challenges From Participants Around Implementing and Engaging With the DCP

Participants identified possible challenges around implementing and engaging with the DCP (Box 6). Both staff and patient participants recognised poor digital literacy and time constraints as potential barriers to engaging with the DCP. Staff participants also identified additional barriers to patient engagement, such as low health literacy, survey fatigue, low motivation, lack of human contact and lack of follow‐up provided for patients who complete the pathway. Two staff participants perceived possible additional administrative burdens upon introducing the DCP. One of the two participants was also concerned about further clinical burden.

Box 6 **Potential barriers when implementing and engaging with the DCP.**

“you're busy working and then I've got my parents who are sick and I go to take care of them and the day doesn't finish you know for me to relax till about 10 o'clock at night. So, you know, it can get pushed aside” *P6*

“Well, I think IT literacy is one barrier.” *S5*

“it might be a bit annoying for them … they've already talked to the clinician or the nurse or the video or whatever, about their back pain and then they get a text message to ask the same questions, that might be a bit challenging.” *S4*

“Using digital removes the human side a little bit further and they might feel less listened or appreciated.” *S7*

“The biggest and most obvious [challenge] is just language and health literacy. Engagement. Perceived, I guess the benefit of ‘what am I trying to get out of this?’” *S2*

“And for the more discerning ones, I think they will question why they're still being contacted when they're not having treatment, but I think that will be a small percentage.” *S1*

“additional time required by the clinician to interpret those results and be able to apply them to improve the way that they provide care. And in this clinic, [we] don't really have admin [support]. So any additional administrative tasks will fall on the clinician as well. So yeah, there the two kind of challenges I see. … time required other than seeing the patients, doing their notes and the follow up to interpret the problems and then to any other additional admin that comes with that would fall on the clinician.” *S1*

### 6.3 Facilitators and Recommendations for Improving Engagement With the DCP

Most participants reported that altruism was the main motivation for patients to engage with surveys. Patient participants reported being motivated to help others in similar conditions and assist with the research (Box 7). However, one staff participant had contrasting views and felt that that may not be enough to motivate patients. Some patient participants also reported being more likely to engage with the pathway if they were tracking their progress and reducing their risk of reoccurrence. Participants also felt that having resources such as videos and personalised results would increase patient engagement with the DCP. Furthermore, participants agreed that having continuity of care through periodic contact with health professionals was important and could potentially improve engagement with the DCP. Staff reported that having an empathetic approach to managing patient expectations may further improve engagement. One staff participant pointed out that patients would respond positively to short waiting times for clinic appointments.

Box 7 **Facilitators for engaging with the DCP and suggestions for increased engagement.**

“I've been obviously doing [exercises] everyday because I don't want that pain” *P3*

“That way, I can also look back and see it, too, whereas at the moment, it's in my head, that I know it's getting better but if it's all collected by you, and I can see it, I'm like, Oh, look at that [graph]” *P4*

“I think people in general are motivated to help out others. And so I think as long as it's in a usable format, and people understand how the data why the data is being collected. I think I would hope that it wouldn't be too much of an imposition of them.” *S4*

“I think videos are a good move, and short is in the right direction” *S1*

“So I think in terms of the expectation video, personally, I think that … that's very important in back pain as I've already mentioned.” *S4*

“I think that engagement during [the DCP], someone's calling who cares, who knows what they're talking about, and offer them good advice about how to get better? I feel that will be a great motivator.” *S5*

“You know, like I said, even you know after a week or so, and then that's it stop until comes again, but who do who do we contact, … what to do when we have the pain, you know?” *P8*

“… I guess the thing that's tricky is it's nuanced for every patient who comes in … blanket reassurance isn't nuanced enough to capture the doubt that people have, that you've misinterpreted their symptoms. And you've misinterpreted their underlying complaint.” *S2*

“hopefully modifying expectations is a huge issue because I think a lot of the back patients that I see have unrealistic expectations that we're going to fix them. And the first thing we have to do is that we'll try and say we're going to manage this issue or we're going to try and help improve …” *S7*

## 7 Discussion

Current clinical practice guidelines recommend for people with low back pain to be managed initially in primary care; however, low back pain remains a common reason for ED presentations [13, 29]. Acute or acute on chronic low back pain can be unbearable for people, adversely impacting their function and quality of life. People may seek urgent care due to a fear of an underlying serious pathology [30] or because of disability and functional limitations [31]. People often perceive ED as a place where they can receive rapid pain relief [31, 32] and advice on how to manage their pain [32]. In some cases, people are not able to access services easily in the community and therefore perceive the ED as the ideal place for immediate comprehensive medical care [13, 30, 32]. This expectation contrasts with the reality of ED, where low back pain patients are unlikely to be admitted and are discharged to the community for further follow‐up with their primary care provider [32]. This expectation discrepancy can lead to poor patient satisfaction and unnecessary ED presentations [32]. Similarly, in our study, patient participants linked poor communication and education with negative experiences, whereas patient participants who had sufficient information felt that their expectations of the ED presentation were met. The fast‐paced, high‐pressure nature of the ED environment often results in limited communication and education from staff [13]. ED staff face service‐level barriers such as ED bed capacity pressures leading to a focus on discharges, potentially resulting in less‐than‐ideal patient care [13].

The introduction of a DCP as an adjunct to usual practice could optimise care for patients who present to ED with low back pain. Our findings show that empowering patients to manage their back pain is valued by both patients and staff. Vaillancourt et al. [33] reported that patients who attend ED want reassurance about their condition, education around the cause and expected course of recovery, symptom relief and ongoing management plan. Similarly, participants from our study highlighted that the DCP could provide education and reassurance, and inform them about expected trajectories around recovery and return to work. DCPs have been introduced effectively in oncology, where they have supported patient self‐management, improved their quality of life and provided reassurance [34]. Research has shown that people with low back pain often continue to experience pain and disability up to 6 months post‐ED presentation [12]. In some cases, this ongoing experience could result in patients seeking further medical input. A study of 14,000 ED presentations reported that approximately 0.9% of people with low back pain re‐present within 48 h of initial presentation [35]. The provision of education through the DCP could reassure patients and manage their expectations around symptom progression, potentially reducing the likelihood of representation to ED. The learnings from the study will be used to develop a DCP for back pain patients presenting to ED [19].

Optimising acceptability is important to ensure that the health intervention is effective in the long term and that end users and deliverers will implement the programme [36]. Understanding stakeholder preferences is key when designing and developing new care models. Engaging consumers and service providers will ensure that barriers can be identified and addressed to ensure effective implementation [37]. Challenges that were perceived in our study were in line with challenges that have been faced in digital solutions for oncology care. These included limited digital and health literacy, and time constraints that impacted on implementing and engaging with digital solutions [34]. Another barrier that was raised by staff participants in this study was the lack of contact with health professionals. In a study by Austin et al., a remote patient monitoring system where patients tracked their symptoms was integrated with the electronic health record and was available for viewing during consultations with their healthcare provider [38]. This integration proved to be helpful for both patients and clinicians by producing a clear overview of symptom progression and supporting shared decision‐making. This highlights that integrating the DCP with patients' electronic health records would improve continuity of care and motivate patients to engage with the DCP. This was also identified as a facilitator by participants in the study. From a health service perspective, however, there may be a disconnect from a volume‐based physical presentation business model and a remote monitoring digital model [18].

### 7.1 Limitations

The findings of this study should be considered within the context of its strengths and limitations. Although some participants in our study had different countries of origin, most had a Western, educated and industrialised background. We also excluded non‐English‐speaking people; therefore, our findings may not be reflective of culturally and linguistically diverse people. Participants who consented to contribute to this study may have a prior interest in assisting research and, therefore present skewed opinions or perspectives around engaging with the DCP, leading to selection bias. A strength of our study is that it captured both patient and staff views, leading to important insights that can contribute to the effective design of a DCP.

In conclusion, patients and staff perceive the introduction of a DCP to be an acceptable measure to contribute to the management of back pain for people who present to ED. Barriers to implementation include lengthy surveys and usability. Facilitators include participants and staff being able to track and visualise progress reported in surveys. The design and development of a DCP will need to consider reported facilitators and address perceived barriers for increased engagement.

## Author Contributions

**Emily C. Bell:** conceptualisation, investigation, writing–original draft, methodology, formal analysis, project administration. **Hazel Heng:** writing–original draft, investigation, methodology, formal analysis. **Nicole Alousis:** investigation, project administration, writing–review and editing. **Matthew G. King:** conceptualisation, funding acquisition, writing–review and editing, project administration. **Andrew Hahne:** conceptualisation, funding acquisition, methodology, writing–review and editing, project administration. **Thomas Collins:** investigation, funding acquisition, writing–review and editing. **Katharine See:** conceptualisation, funding acquisition, writing–review and editing. **Tracey Webster:** investigation, funding acquisition, writing–review and editing. **Elisha O'Dowd:** investigation, writing–review and editing. **Paul Jackson:** investigation, writing–review and editing. **Adam I. Semciw:** conceptualisation, investigation, funding acquisition, writing–original draft, methodology, formal analysis, project administration, visualisation.

## Conflicts of Interest

The authors declare no conflicts of interest.

## Supporting information

Supporting information.

Supporting information.

## Data Availability

The data that support the findings of this study are available on request from the corresponding author. The data are not publicly available due to privacy or ethical restrictions.
