# Supplementary material for: Patient and Staff Insights on Digital Care Pathways for Patients With Low Back Pain in the Emergency Department: A Qualitative Study
Source: Health Expect. 2024 Aug 16;27(4):e14182. doi: 10.1111/hex.14182 (PMC11329376; doi:10.1111/hex.14182)
Supplement: Supplementary file 1 — Supporting information. [file HEX-27-e14182-s002.docx]

**Interview Schedule Patients**

Introduce staff

This interview will take 30 minutes. The information gathered will be used to design and implement a digital care pathway for people with back pain presenting to ED. This research project has been designed to ensure the researchers interpret the results fairly and appropriately and avoid researchers or participants jumping to conclusions.

Within two weeks of completing the interview, we will send you a copy of the transcript of your responses. This is an opportunity for you to check your responses and provide any edits if we have accidentally misrepresented anything you said. We ask that you complete this within two weeks of receiving the transcript. If no feedback is received after two weeks, we will consider that the transcript is correct.

- - Tell me about your experience with back pain?
  - What treatments have you had?
    - Probe: Any experience with digital care pathways?
  - ***Demonstrate current model of digital care pathway***
  - What outcomes are most important to you?
  - What are your thoughts on the model?
    - How acceptable is this model?
    - What challenges can you foresee?
  - We will be using technology, how engaging would it be to receive information about your care through:
    - - Email
      - Text messaging
      - Mobile applications
      - Video calls
      - Other?
    - Do you have a preference and why?
    - Appeal of patient dashboard- what would engage you with reporting your outcomes? (What would motivate?)
    - Do you have suggestions for additional resources?
    - How do you feel about clinicians being able to remotely monitor your health information? (is it acceptable?)
      - May include your responses to pain questionnaires, routine physical activity saved through a personal device (e.g. phone, smart watch etc)?
      - What benefits might there be?
        - May need less visits to health care professionals?
        - Better communication about your care?
      - What concerns might you have with this?
        - Data security?
  - What other aspects that your healthcare team should monitor?
    - Physical activity?
    - Others?
  - How often is reasonable to fill out questionnaires at home? (How long?)
  - What aspects would make you more likely to engage with remote monitoring?
- What information would you have liked to know about your back pain?
  - Before coming to emergency about the process and managing your back at the time of emergency about your condition or attending emergency
  - After leaving emergency what information would you like to have known about back pain or course of management
  - Before attending the HOT clinic appointment
  - After attending the HOT clinic, is there anything you still have questions about?

**Workshop Interview schedule – ED staff**

Introduce research team

The interview will be conducted on Zoom and recorded. It will take 30-45 minutes of your time to be part of this study. The information gathered will be used to design and implement a digital care pathway for people with back pain presenting to ED. This research project has been designed to ensure the researchers interpret the results fairly and appropriately and avoid researchers or participants jumping to conclusions.

Within two weeks of completing the interview, we will send you a copy of the transcript of your responses. This is an opportunity for you to check your responses and provide any edits if we have accidentally misrepresented anything you said. We ask that you complete this within two weeks of receiving the transcript. If no feedback is received after two weeks, we will consider that the transcript is correct.

- - What outcomes are most important to you for people with back pain who present to ED?
  - Would you prefer paper or electronic records of PROMs?
  - ***Demonstrate current model of digital care pathway***
  - How acceptable is this model to you as a staff member?
    - Would your patients have access to
      - Email
      - Text messaging
      - Mobile applications
      - QR code
      - Other?
  - What challenges can you foresee for clinicians and admin staff?
  - What challenges can you foresee for patients?
  - Do you have any suggestions for operationalising this model
    - Text messaging vs email
    - Appeal of patient and clinician dashboard
