# Supplementary material for: Patient and Staff Insights on Digital Care Pathways for Patients With Low Back Pain in the Emergency Department: A Qualitative Study
Source: Health Expect. 2024 Aug 16;27(4):e14182. doi: 10.1111/hex.14182 (PMC11329376; doi:10.1111/hex.14182)
Supplement: Supplementary file 2 — Supporting information. [file HEX-27-e14182-s001.docx]

Theme 1: **Expectations and experiences of staff and patients with low back pain in ED**

| **Subtheme:** | **Finding:** | **Supporting quotes:** |
| --- | --- | --- |
| People with low back pain who present to ED with severe symptoms want effective communication to set expectations around their ED journey. | Back pain is a debilitating condition that causes feelings of guilt and anxiety. | “…the Friday when I went to the emergency room, I just had the pain all down my left side and down my left leg to my foot to the point where I actually couldn't even walk, …the pain was just so excruciating. I couldn't even function” P4 |
|  |  | “[I felt] guilty coming into the hospital with just a back pain and not life-threatening illness. But I was just in so much agony. I kept saying I'm so sorry I'm here... But this pain was so bad. I was screaming in pain by myself at night because I couldn't sleep flat. I couldn't turn from one side to the other side. It was just so excruciating. And then I …dread going to bed because I knew what was to come so horrible as well.” P6 |
|  |  | “I felt like I was dying right in it. Just standing there like, oh my god, am I gonna actually get to where I need to go? …My whole body felt like, every time I went to take a step, I'm like, oh my god, this is so painful. Like, am I going to be able to get anywhere?” P3 |
|  | Patients want clear expectations about their ED journey. This could be achieved through effective communication and education in hospital. Patients who received information about what to expect reported positive experiences. | “My experience when I went last time, wasn’t that great... The paramedics took me to the hospital and got there, waited 15 minutes and when the nurse came to me, I was on the stretcher. She spoke to me [for] two minutes…You have to go sit in emergency. …my pain wasn't important or not serious enough. …I think this would be more helpful to have you know, when you get there in pain, to have something surrounding the patient… Yeah, yeah. Because from my last experience, I'm like, if this happens to me again, I'm not going, I'll just take my painkiller and stay home... [I] didn't even know that [I] will have this follow up. You know, I will be taken care of by a physio… [at the back pain] Hot clinic. …I went to the clinic but you don't have any information from the beginning. I thought it would be less like this, you know? …Yeah, they didn't even tell me the details of painkillers…no advice like do this exercise, this will help you go. Yeah, was it a lot of things missing? Maybe just not even- they didn't even- I think they gave me just some strong painkillers other than that just one but didn't give me anything [else]. So everything was missing on that night.” P2 |
|  |  | “…everything that was explained to me at the hospital I thought was fine. I didn’t feel out of the loop or anything.” P5 |
|  |  | “...You'll be given painkillers. You know, someone will make an assessment on you. Am I having a scan tonight? Am I staying overnight? Just to also make it easier for my wife to sort of plan around” P7 |
|  |  | “Because I remember [a] couple of days later on, because I was probably still tired and emotional. I remember crying to my son going, you probably just think I was overreacting having to go to the hospital.” P4 |
|  |  | “I think I knew what to expect when I went to [the hot clinic]. They explained to me that they are sending me to the back pain clinic. Someone actually called me as well about it. So I think that information was provided really well. …And in the back pain clinic that person was really, really helpful as well. The physio there and …the entire experience was really, really great and I really appreciate it.. [I] think the hospital is pretty amazing. You know, they really helped and took care of me in both those cases” P6 |
|  |  | “I tried at least walking around as much as possible. But …what if I'm at home… I'm just sitting there constantly taking painkillers. …is that going to help me in the long run?…in the hospital if I was told, like, just by sitting in the chair, doing the pelvic tilt or laying in bed, do the pelvic tilt. …maybe I wouldn't have been in there as long and the pain would have subsided a lot quicker…Yeah. He just said take a pill. Right? Not like what might have been better if they said ‘try even some sort of simple exercises like, okay… try doing this when you're sitting down’ like I was, when I went into the into …the clinic after I came out of hospital, where I was given …the pelvic tilts. If I'd been told that in the hospital, …maybe the pain wouldn't have been so constant.” P3 |
| Outcomes that are important to patients and staff  Patients and staff have overlapping priority outcomes and staff report additional job-related outcomes.  Patients and staff share similar outcomes however staff acknowledge additional challenges when delivering patient care  Patients and staff focus on similar outcomes  While staff outcomes are influenced by additional pressures | Patients and staff report similar important outcomes for patients including pain management, quality of life and mobility. | “I want to achieve a full recovery. …that I can conduct any physical activities …without fearing or thinking in the back of my mind, if I bend over here is my back going to go out again, or …that if it does happen again, it won't be as painful as it is now. …I don't want to be restricted in certain activities. …I don't want to stop playing golf for instance or doing something active like kicking the footy or playing cricket.” P7 |
|  |  | “…to be able to live without pain. …just to be free to move …don't have to think twice before doing anything… Yeah, better life.” P2 |
|  |  | “…the outcome I'm mostly aiming for is getting people comfortable, but also with the multimodal approach to their pain and their sensation of pain to try and allow them to be at home.” S4 |
|  |  | “If I'm being patient focused, it's obviously to provide reassurance and education and adequate pain relief, and a plan post to discharge for the patient so that they leave comfortable, that they know that they that their severe pain is not life threatening and that they know what to do. And who to go to.” S6 |
|  |  | “…pain you know, …that it will be easy …this is first priority to me …and, obviously, …I do like exercises or [going] out or …gardening...Getting dressed...Maybe working” P8 |
|  |  | “…how are [people with low back pain] feeling when this sort of pain occurs” P3 |
|  |  | “…how are they going in terms of getting back to their function? Or what's the quality of life like? …whether that be sports and fitness and getting back to the gym or whatever they were doing previously, or just their general daily activities or like we're an older patient. Their ADLs and whatever.” S2 |
|  | Staff report additional outcomes, including the ability to discharge home, red flags, and education. | “I think education for patients, it's pain relief. …to communicate the fact that they're not going to they're not going to damage their back by doing things. …If you pick up something from the floor …it might cause pain. …there's a difference between pain and damage.” S3 |
|  |  | “…we don't always have time in emergency departments to be honest to do that. But the education side of things is important. And it's also the communication log going hand in hand with education… But from an emergency point of view and a hospital point of view, it's also the investigation. …There's certain circumstances where scans can be warranted, but most of the cases that will come through the door, don't need a scan that day.” S7 |
|  |  | “So from an analgesic point of view, enough to mobilize and be able to ideally return home under the same day to avoid keeping them in hospital and some of the things that can happen as a result of staying in hospital too long. Secondary kind of illness, lots of imaging, lots of Bloods, lots of communication. All of those types of things are avoidable if we can get people comfortable. And yeah, and get them home.” S2 |
|  |  | “My back pain, what is causing my back pain? And how soon can I expect to get back to being pain free or back to getting back to work? It's really those who are obviously of working age. That's, I think those are three big questions.” S5 |
|  |  | “…making sure we're not missing serious pathology, and then patient expectations. And then kind of assessing level of function ability, so mostly aiming to get people home. I don't know that keeping them in our hospital really helps a lot of them.” S4 |
|  |  | “That they feel confident that they have been heard and that they have a plan for moving forward and getting better and that they feel like they've got an understanding of what they could do when things don't go to plan.” S1 |
|  | ED staff face competing interests and challenges when providing evidence-based holistic care. These challenges include organisational pressures, time constraints and managing patient expectations. | “…what I see and this is also problematic, is that people they often will get admitted for back pain, because they're in pain even though they can walk and they often get opioids and they often get an MRI… in the end you have to get patients out your department. …I feel conflicted about it, especially because in the past, I think it was much more of an emphasis on minimizing intervention, whereas now there's more emphasis in emergency on getting the patient out. …I want to shift… towards other things, you know, like, exercise or even, you know, like posture when they sit or standing or walking, weight loss and things like that. But it's hard to do when people are in acute pain. …We need to remind the doctors- I think everybody was trained in the general sense of…trying to minimise the need for imaging, minimise the need for pharmacology. But if at the same time there's a four-hour deadline, then it kind of takes the- your decision making is just because of that, even though you don't want to, but after a while, you forget because you're so used to… giving them opioids, sending them to the ward and then out of your mind, of your sight. Everybody's happy if the patient is happy. You know, your bosses are happy because the department- the flow is good.” S3 |
|  |  | “…I wonder if there's a discrepancy [in] care day to night… there's no consultant working in fast track. So I have observed that the care is extremely variable… I wonder if there are patients that do get scans on different days depending on who's working.” S4 |
|  |  | “My role in the emergency department is to do those things for the patient but also in a very timely manner, and to discharge wherever possible and not admit wherever possible.” S6 |
|  |  | “frankly speaking, the most important outcome for me is whether I can get them home and out at the emergency departments in a timely fashion.” S7 |

Theme 2: **A digital care pathway can empower patients and support clinicians in providing care**

| **Subtheme:** | **Finding:** | **Supporting quote:** |
| --- | --- | --- |
| Participants anticipate benefits being associated with the introduction of a digital care pathway for people with back pain | Patients view tracking personal progress and benchmarking against aggregate data as valuable for managing symptoms and expectations. | Because for me, it's also would be nice to know that this is actually normal, even though the doctors had said so it would also be nice if there was some data showing. This is normal and I'll get the feeling back in my foot at some point. …Oh, quite important, maybe a good idea of when you should return to work. So you kind of have an idea because I mean, I took one week off and then I was back to work more mainly because I just couldn't afford to take another week off work. But I also didn't know whether that was okay to go back to work or not to go back to work. So sort of indications on when it is safe to because I kept asking the doctors is it alright if I walk on my foot, even though it feels weird, and you know, just knowing what is the best thing to do for your body?” P4 |
|  |  | Well, I've had this flare up at this time. And then like, track how many flare ups that occur. … Like how often it's actually occurring and when they do when it does flare, were you doing something strenuous or where you're just sitting on a chair relaxing?... And the timeline, say, ‘Okay, well, it started on this date. Then it ended on this date.’... Sort of like, then it can like give people a lot a lot clearer... maybe I can manage the pain for that amount of time if it's going to be really bad at this point... like give them a better mental picture of, okay. It's only going to be three weeks, like I'll be better in three weeks time or whatever. … That would be great, that would actually give them a lot more knowledge that they can pass on to others as well. Okay, in in this study we found this like, and then integrate that into that person's care, maybe like they will feel better, a lot quicker, or like at least have the idea of okay, I don't have to stress about too much. I've got the light at the end of the tunnel. … I think just the more knowledge this person has to help combat their pain and get them back to normal. And just integrating it into other people's care. So, this is what the study of has shown us. Let’s keep the medication to sort of like in a way a minimum. Because I know for me, I hate taking medication if I don't have to take it. That okay, these are these are alternative methods to use instead of the constant barrage of medication... I think that [tracking outcomes] is so much better. Because like, with all even the surveys, I can say okay, well, if this is done to begin with, like maybe like there'll be less medication having to be taken more. More sort of first-hand knowledge of going well okay, doing this is better than doing that. …Yeah, [graphs] would be good because then I can say, Oh, okay, the pain decreased because I did this extra today.” P3 |
|  |  | “Yes, predictions. Yeah, definitely will be good to have for [returning to] work also. Yes, it will be helpful, it's my responsibility also to be able to be back to work as soon as possible. And to get because I have to answer to, to my boss, my bosses also. So yeah, predictions will be very important.” P2 |
|  |  | If it displayed a graph that said, ‘your pain was here and now it's here’ that definitely would [be helpful], because that's really targeted to me... Yeah [predictions for returning to work and being symptom free] definitely. Because they're for you personally.” P6 |
|  |  | “You'll find [graphs] engaging. I do because I've got something similar at the moment with a sleep apnea machine I've had for about four or five years. There's an app and initially… I was on the app every day just to monitor my sleep patterns. And it provides graphs and data and statistics and all that on my improvement of using the machine. And within a few months, I completely disregarded the app on it because I could feel the improvement already and I could see the data was in front of me. So all that information is relevant especially initially, to improve your health, obviously. It's also a good reference point. I might go on once a month to check my sleep patterns, so forth. If I'm having an issue sleeping, I'll go and examine the data of the last week or two and say, ‘Okay, what's happened here? Why am I getting up more frequently? Or is there something wrong with a machine and or, you know, there's something wrong with my mask?’ It's a good reference point. So yeah, great information.” P7 |
|  | Patients felt that having a health professional monitor their back pain journey would be beneficial. | “Well, they could maybe make an assessment so somebody's on top of exercise isn't exercising enough, or they’re exercising too much. They can make an assessment and they say that contributes to the pain.” P5 |
|  |  | “Just continue to have that care and support this back pain is an educational I would say, and a connection. I think the biggest thing would be the connection because I feel very isolated and, and alone and don't know much about this thing that's just suddenly crept up on me. So to have that connection with, even if it's not someone specific like yourself, I still have I'm sure somebody will be responding to me right, as a response and stuff. So I would feel like I'm not alone in this journey.” P6 |
|  |  | Well, this is the first time [having remote monitoring] so I haven't tried it before. Let's give it a go, you know? P8 |
|  |  | “Yeah, but it would be nice. To have you know, a trained person actually monitoring and, and knowing that there is improvements, and they can see the improvements.” P4 |
|  |  | “…I suppose if they're monitoring my wellbeing and if they feel like perhaps I'm lagging in a certain part of my recovery, they could notify me I suppose, or, you know, if they feel like I'm not exercising as often as I should, to help with the treatment of my injury. That'd be a nice prompt, I suppose. That'll be beneficial. And I suppose if they do collect enough data over a period of time and they can sort of see a general improvement to my condition, or if there's a lag in my improvement, they can sort of okay, let's identify the root cause of this you know, am I exercising correctly or am I not exercising? Am I not? Following say a schedule, you know, and had returned to full capacity from my injury. So, if there's a program not falling correctly or not following the rules, that'd be probably the only benefit I suppose leads to significant benefit anyway.” P7 |
|  | Participants agree that remote monitoring may reduce physical visits to health professionals | “there's more points of contact between the healthcare provider and the patients. I think it makes them feel more validated, maybe makes you feel less lonely. I think it may give them a sense of comfort and justice with pain isn't just the pain itself.” S3 |
|  |  | “I think that will be really good. Because for me personally, I don't drive a lot. I do have a car, I drive in my local area, but to get to Epping– I've been there twice now– I had to get a taxi back to and from even though I live in [location]. For the type of work that I do, I am a learning specialist so I train people. So I'm like involved full time during the day. So this would be mean that I can actually do it on in my own time.” P6 |
|  |  | “if I am looking after myself, and I'm following the correct advice that's been offered [digitally] to minimize any additional injury. Yeah, I suppose it would be beneficial to just a matter of being disciplined enough to follow those guidelines that have been offered [to avoid physical visits].” P7 |
|  |  | “to ease the pressure on GPs and, and hospital you know, emergency hospitals and all that things, you know, if I could manage, you know, the pain. I wouldn't call the emergency department and go there.” P8 |
|  | Staff viewed the DCP as a way to manage patient expectations around back pain and predictive data for clinicians about representation and chronic pain. | “I think [the DCP] gives us peace of mind that you I mean, part of us doing imaging is also not missing. Serious causes of back pain as opposed to the sort of standard, you know, muscular skeletal muscular, fascial back pain. I think it's good. I think I can explain to people, yes, they're going to be in pain. It's going to last for you know, a couple of days a week or two, but then it gets better. And then where to go next. So explaining what to expect. I think people know that but what's going to happen? They're more accepting of their pain in a moment.” S3 |
|  |  | “I wonder if it would be useful for clinicians involved, like I think, if you could give data to patients saying, you know, we've collected data of 30 patients and you know, 75% of them are better or feeling better at six weeks. I think that's positive hope for someone. Everyone assumes they're going to be in that 75%. I love that data. I think it's really powerful for patients to say, you know, like, I think in shared decision making and explaining illness trajectory, I think it's really helpful. To say, you know, this is how most people recover from this or this is how most people experience whatever it is, you know, like, yeah, 20% of patients go to the back clinic and don't find it useful, but 80% Do or whatever I think it's like, I think that's very useful. Data, especially in something like back pain that we don't have that many therapeutic interventions in emergency for. I think being able to help set expectations with that data is very useful... Again, I think it really strongly depends on a patient's health literacy. So I think if it was me, for example, was suffering with back pain. I think if I could see someone similar to me, whatever similar age or, for example, who got better in six weeks through whatever process it was, I think that would be a positive affirmation that you weren't going to get better.” S4 |
|  |  | “I think [data from the DCP] that's so important because people expect tablets. I guess in the acute phase, that's really important. And you're right at that point, they're not going to listen to anything. That's not helping them immediately resolve their pain. So I think that you can talk about it… in a timely manner, which is acute back pain. You can expect some, tablets, they may give you some stretching or strengthening exercises, or you know, and then or you know, you need to rescue me, and I said, and then after 72 hours, you can do some gentle stretching, mobility and you know, I guess longer term, it's about fostering learning and all that so, yeah, I think that's going to be important to, to sort of educate, I guess.” S5 |
|  |  | “And the other thing I suppose, is that that solid graph from our point of view is going to help identify somebody who may be is beginning to drift into the chronic pain zone or the regional pain or something along those lines where you need to be aware of that as well. So that's a tool that can be is not necessarily negative in that it just shows that what's been man done so far is not working, and it's consistent with what the patient's reporting...knowing that a number of episodes of back pain will kind of resolve it in six to eight weeks regardless of what you do. The evidence tells us that maybe this is enough...Definitely, I think something visual like that a little bit of a reward sort of thing or a feeling of you know, progress or tracking the progress and also that they can see us and then that they know this, the physio following them can see us and so this, you know, they're able to, even if they, if they don't necessarily feel it at the time, when they retrospectively track it, they can see that, you know, well actually, you know, you said the pain was you're saying it's six out of 10 But you know, it was nine out of 10 when you first came to us and then eight and then seven. So while it's not getting better quickly, it is getting better things like that could be definitely useful for them to feel that it's been tracked.” S7 |
|  |  | “The thing that's tricky, from our point of view is we do really want to be collecting routine data that we can kind of compare these patients against and also stratify, in particular, their risk of re-presentation and chronicity of complaint.” S2 |
| A DCP could improve patient care by empowering and supporting patients through education and resources | Participants agreed that reassurance and education around returning to usual activities would help empower patients to manage their back pain. | “Giving [the patients] some reassurance and timely education messages around that pain ... in that recovery time, reassuring them of you know, appropriate, helpful messages that may have been started for the first time in ED. …we want to get across in reassuring patients that it is safe to move. Highlighting to them that some gentle exercises and progressive exercise as appropriate can help and that that's a treatment.” S1 |
|  |  | “I think things like, what the hell do I do after I go home in terms of, is it hot packs or cold packs? Should I sit in this chair or this chair? Should I be lying on my side or on my stomach or on my back? When I'm resting how often should I get up and move?” S2 |
|  |  | “...it would be interesting, I don't know if it'd be involved in like the general, like kind of gentle exercise and expectation video, ... and physio and exercise and rest and, you know, whether it be heat or cold packs, depending on who you ask and stretches and things like that.” S4 |
|  |  | “I think it's really good to normalize it in the you know, the procedure. And also I guess will be helpful in that video is the expected recovery... So I think like having a timeframe might be helpful to manage expectations... for videos around how to do things, how to pick things up, to protect your back and things. I feel like we're so involved in the security, curative space or therapeutic space when there's so much to be done in the prevention, space, and ownership of their health.” S5 |
|  |  | “If I'd been told that in the hospital, like maybe the pain wouldn't have been so constant... if I continue doing the pelvic tilts or if I maybe sat a different way or I exercise more slowly or more often. Maybe that could help with the pain then I'm not constantly going into hospital to have them try and deal with the pain. …doing other things that could help with the back pain. And like, just getting a lot more information about different strategies.” P3 |
|  |  | “I think reassurance is a big thing that, yeah, you're not alone. And you're not, you know, crazy or overreacting for going to the hospital in the first place. I think it probably would have made me feel a little bit more at ease knowing that it actually is such a big problem and one in five or one in six suffer from it and present to the hospital….[It would] be nice to know the statistics when you get there that it is quite a high thing to go to the hospital for and you're not maybe overreacting and then yeah, that there is going to be help available to you after.” P4 |
|  |  | “I understand, of course how busy [ED is], I think this would be more helpful to have when you get there in pain to have something surrounding the patient.” P2 |
|  |  | “I would absolutely appreciate it because that's just like I said, I had forgotten the exercises that I was supposed to do and I wish I could recall them. You know, so that I’m on the right track with the digital pathway. Education and support. Yeah, so that I think would be a really good way to continue because right now the pain has receded. But yeah, gone away. So there's been no proper fix. So it'll be continuous if you do this, and that for me personally... there was that assurance and that little bit of education as well in how to live with this back pain... I guess maybe just some education in how to manage back pain. …and how you said little things like, one in six people have got back pain. Good little tips, kind of like flashes of things like continue to walk or go to a physio or a swimming pool, or take that pain medication before it gets to you... even positive affirmation, saying like, you're not alone or let's work together in a little group or I don't know, it's just all those things. Because the moment that I started telling myself, I should get a dog, the pain started to ease off even by that so sometimes it's just people who live alone, wintertime. It's just nice to have those little affirmations and things like that as well. I think spinning whatever you're seeing in a positive, we're not like just telling you what to do. If that kind of be relatable to you.” P6 |
|  |  | “how to deal with the pain and how to deal with the things you know, so an impact on my life.” P8 |
|  | Patients lack an understanding of indications around imaging. Staff recognise this and therefore want to manage patient expectations by normalising evidence-based clinical management. | “Highlighting that even if scans are not required at the moment that that that's not something that's permanent, and if the situation gets worse than that can be revisited and empowering them to understand what are concerning signs and symptoms that justify coming to an emergency department again... Setting up the expectations and this is part of your assessment with the specialist back pain clinic where they assessed if you need imaging, you can talk about that with them whether they assess if you need any specialist referrals most patients in this catchment are impressed by someone taking the time to see if they need any specialist referrals and being able to follow that up and explain to them the timeframes in which that is required.” S1 |
|  |  | “the videos surrounding imaging needs to be approached really carefully because if you put a list of red flags, that is while I guess you're a trained professional, it might make a lot of sense. There's a chance it might instill fear instead of reassurance….normalizing the fact that a lot of people don't actually need scans, because isn't going to change our management... Normalizing the fact that actually you do not need imaging, or like you know 70% or whatever the figure is to go with that is actually muscular pain or you know, it's not dangerous” S5 |
|  |  | “Yeah, maybe how much they're [imaging] actually needed? Because I had, first I had an ultrasound and I had an x ray. Then I had a CT scan. But when I saw the [physio] at the back clinic, she said that none of them were really necessary in the beginning. Yeah, and she picked what my problem was just by my symptoms, and the test she did that day on me. She knew exactly what it was. And she told me what this cat scan was going to tell me. And so I had three tests that I didn't have” P4 |
|  |  | “I don't know really, knowledge of X rays, but, you know, a call from the medical professionals that you know, and I'll just take their word for it. And I got showed some pictures and I was like, get like a but like, I don't really? I guess I'm not an expert in that area. So yeah, try to take a word for it.” P5 |
|  | Participants indicated that education about common pharmacology for back pain would be helpful to empower patients to manage their analgesia independently. | “And the analgesic information that we give people we throw a whole range of new medications to people on a day that they're in crisis and often drugged and then wave them goodbye. And expect them to remember that they should take two paracetamol every four to six hours, no dramas where they've taken food or not, but the ibuprofen is four to six hourly, also with food. And then on top of that, they can take one Endone tablet. Like it goes on and on and on. And we expect that pharmacists give good education around those things, but to be honest, that's really kind of passing the buck when they're also under resourced. …I guess I always argue I would always start to I would always argue that yes, there is really very real legal implications for education, merging in with prescription like, essentially, if you start to write down for people on paper, to take things at particular times you're then really ultimately you've given them a prescription.” S2 |
|  |  | “multimodal care and like you know, kind of simple analgesia... I think that's useful.” S4 |
|  |  | “I would believe that, like just knowing that, okay, people, like medication might not work for everyone” P3 |
|  | Staff want to manage patient expectations around surgery, highlighting that surgery is not indicated in the first instance. | “some people expect surgery for some strange reason, right, they associate in correcting back in surgery. Whereas with, you know, a lot of these bulges that's, you know, strangely enough, the bigger disc bulge the more responsibilities to conservative management... even a video addressing why surgery is not the first choice.” S5 |
|  |  | “We know a lot of them don't need are not surgical candidates anyway. I think they're trying to do a similar thing with back pain. And because I think a lot of people get referred for surgery that don't need it for back pain.” S1 |
|  |  | “Other people come in, convinced that they have to have some surgery on their back. And, you know, I'm not convinced about the role of surgery. You know, full stop, unless you've got something very clearly problematic. And I mean, there's been stuff before out of the US where they looked at this going back years where they were talking about all these discectomy infusions that were being done without a huge amount of evidence to support it, but when you start introducing something like that, people then assume that that's how their back pain. So that's, it's all about modifying expectations, so that people and I mean, it's not just the patients because I get people sent in from GP’s who are coming in saying ‘the GP said you would do an MRI and the GP said, I probably need surgery’, and so on. And you have to modify that as well.” S7 |
|  |  | “…definitely surgery isn't the answer for this, which a lot of people kind of are hopeful for.” S3 |
|  |  | “One way thinking might include just some basic reassurance that back pain is fairly common, and in most in a lot of cases, you don't need surgery.” S6 |
|  | Three participants indicated an interest in resources linking to alternative medicine and psychological services. | “...every hospital I've worked at has had their own kind of handout or is used a different handout, but they've all said the basically the same things. I'd be pretty interested in if you guys were going to include some like recommendations for mindfulness or meditation apps, like I think there's a lot of room for people to use them for pain modulation... So I wonder if that might be like a little section about with psychological support and ways to focus on other things.” S4 |
|  |  | “Even alternate treatments, not just physio or osteo, or things like that. Because I had someone tell me about this lady that's called a manipulator in Heidelberg. And I never knew anything about that until someone told me so just some other alternatives to just your general ones that the doctor will tell you. …Um maybe some recommended websites to go to because I've looked at I've seen since having my back pain, there's so many things on like Instagram and YouTube and all those sort of things to do this exercise to back pain and do this exercise for back pain. And there's so many and I don't know which ones to draft. Good. Yeah. So maybe like some trusted websites to go to that these ones are the ones that you should be doing.” P4 |
|  |  | “Everything is it's all about multidisciplinary and multi-pronged and multifaceted and so sometimes it's you know, something like that would suggest that may be bringing in either psychiatry or psychology into the management service, maybe may play a role in long term and chronic management.” S7 |
|  | One staff member and one patient reported contrasting views on the importance of dietary advice. | “Dietary advice at that stage is probably not one of the things that I think is particularly important.” S2 |
|  |  | “I guess weight would be really important to make sure that the- because weight plays a big part, the diet, mental health.” P6 |

Theme 3: **Acceptability, barriers, facilitators, and recommendations of engaging with a digital care pathway to track the trajectory of back pain**

| **Subtheme:** | **Finding:** | **Supporting quote:** |
| --- | --- | --- |
| Perceived acceptability of DCP delivery mode, frequency, duration and security. | Participants all preferred electronic delivery such as texts or emails, however there were differing views on which were more appropriate. | “Electronically sure, because I lose everything on paper... I reckon text for sure. Email I feel like…most people will, but I feel like that could be trickier for some.” S2 |
|  |  | “stock standard email or website? Yeah, I can't think of anything more engaging than that, that's gonna be practical.” P5 |
|  |  | “Electronic I think... Just the logistics, I think if somebody would send me a text message saying …rate your …whatever, and you just click a button and it's done. I think you're more likely to get that information... a text message with a like, click on a link... many people don't answer their phone unless it's somebody they know because of spam calls. Whereas a text message- I think it's easier to send and also easier to read and to open” S3 |
|  |  | “electronic I think. …if it's older generations, it might be slightly more challenging, but I think if we've got multi, long linguistic and culturally diverse access, I think most people are used to doing forms online, think it's quick and most people almost everybody has a phone these days. …that's quicker from a patient satisfaction point of view. I assume also much easier from a data collection point of view. …digital is where we should be going for everything. Also, climate point of view” S4 |
|  |  | “Paper definitely not. … Maybe emails for me. Video calls work. Apps, maybe people don't go on phone every time and as they turn on the notification” P2 |
|  |  | “by phone because I don't have a computer” P3 |
|  |  | “Email is better. I think, you know, if I had to choose one, email would be better because sometimes …it gets hidden with so many other messages that come in for work and so on. But with an email, there's a link and so on. …I guess the app would be good because that's where I'm assuming that you will be sending the exercises and things through. So if I have to just download an app and use that, I'll be okay with that too” P6 |
|  |  | “Or through email is easy. We can, you know, communicate… easily…” P1 |
|  |  | “probably email …and just you clicked on a link or directly to a website? Or even if perhaps you just send me a video clip or something or click on something and watch it or view it. But yeah, links through email [would] be probably more beneficial for me. …Email, text, doesn't really bother me as long as there's a link there or get access to your program.” P7 |
|  | Common acceptable frequency of surveys was fortnightly while ideal survey duration would be under 10 minutes. Staff agreed that surveys should be short in duration to minimise burden. A patient participant suggested for the timing of the initial survey to be sent close to the back pain episode. | “I think the longest questionnaire I've filled out for something was probably about seven to eight minutes. …weekly, fortnightly [is acceptable]?” P3 |
|  |  | “…rather than trying to think back like I know how much pain I was in that day but to try and put it into words now is hard. But if I was doing it at the time, I might have been able to sort of articulate it better on how I was actually feeling that day. …at the time that I was in the waiting room of the hospital, if I was trying to fill out forms and watch videos, …I was in so much pain. I wasn't concentrating fully so it might not have been sort of taken in as well as when I was feeling better. …probably once a fortnight, once a week, probably at the most because I think people would just forget. I know, I would have a habit of reading and go, Oh, yeah, I'll do that in a minute. And then I'd forget so maybe, at least once a fortnight, I think is reasonable. …We probably wouldn't go for probably more than 10 minutes. I think that's probably a reasonable time to sit and fill in some information and not sort of think Oh, got another page.” P4 |
|  |  | “Well, I usually sit for about 30 minutes then after I’ve got to go and get up and stretch. So I'd say 30 minutes on the questionnaire is could be quite draining.” P5 |
|  |  | “It's one of those quick ones like you said, you know, how's it going out of one from one to 10, I don't mind if it just pops up every now and again. Want to know specifically? So let's say once. But if it's a long questionnaire, then maybe once in three weeks, once in four weeks... timewise to complete the questionnaire? Yeah, oh, maybe five, six minutes” P6 |
|  |  | “I wouldn't mind spending at least maybe 10 minutes in the morning and maybe 10 minutes in the evening [on activities and education]... On a weekly basis, I can see the gradual improvements.” P7 |
|  |  | “Very good idea within two weeks, that should be improved” P8 |
|  |  | “it needs to be a simplified or time limited survey that doesn't impede on engagement...” S5 |
|  | While some patients had concerns about data security, most patients were realistic about the increased prevalence of hacking and were not specifically concerned about their health data. | “…I trust if anything happens, I know any company will take the responsibility. Many industries, countries …works [like this].” P2 |
|  |  | “As long as everyone tries to keep all the information secure, that's the best that we can do right now.” P3 |
|  |  | “These days with getting a text message and say, click on this link and that sort of thing. If there was an actual app that you know, is the legitimate Safe app, it's probably easier to use and safer. … as long as you don't have these random people, reaching out and trying to get information or feeling and things like that, that's probably what people wouldn't want to happen.” P4 |
|  |  | “Depends on how sensitive information is you know, just my responses and pain questions I wouldn’t be concerned but for all my details, with my name, address, and all those other personal details are big concerns” P5 |
|  |  | “It's going to healthcare professionals, it's not going to anything else. So I don't think I need to worry about that. …You know, the hacking can happen anywhere. …you got to put your trust in things and this is a medical space. So I mean, I got hacked when that Optus thing happened, right? With just a phone service. Buying things on eBay or wherever, right? Your data is everywhere. So I don't live in fear. But if something happened, then you just have to work with it.” P6 |
|  |  | “in the world nothing [is] safe. So this will help people, I don’t think this will affect our life, you know. So that's why I'm not concerned about the security thing.” P1 |
|  |  | “I've got no issue with that and just for research and all that. A privacy issue, you know, that might be concerned about, but I suppose if they're monitoring my wellbeing…” P7 |
|  |  | “lately we've been hearing about this hacking. In all things that's scary to me, you know, it's really scary. You feel sometimes unsafe. And, I mean, it's a lot of things, that may happen, or they may have an access or somewhere, especially an insurance or whatever...just [sharing the information] between my GP and my specialist, that should be enough.” P8 |
| Perceived challenges from participants around implementing and engaging with the DCP | Both staff and patients identified poor digital literacy and time constraints as potential barriers to engaging with the DCP. | “Time taken, like it's hard enough to get them to the appointments, let alone getting them to fill out any extra information. …if they've got time or the inclination to fill it in, they will. If not, they won't. …[if] they don't do it fairly soon or timely, I think it's less likely that they will fill it out at all, even if it's paid … accessing digital resources …just instructions to click on a link will be challenging for some patients. For you know, a number of different reasons, one could be their confidence with technology.” S1 |
|  |  | “it'll be like a 10 or 15 minute survey and then I don't like to start it because if I start a survey I like to finish it …that becomes annoying if …it says to you right up the front this is going to take 10 minutes to complete. Fair enough, you're not going to start it …because you're busy working and then I've got my parents who are sick and I go to take care of them and the day doesn't finish you know for me to relax till about 10 o'clock at night. So, you know, it can get pushed aside and you get one of those bad ones that's annoyed you, you might not open up those again. ... not everybody's digitally savvy.” P6 |
|  |  | “Well, I think IT literacy is one barrier.” S5 |
|  |  | “The only thing I suppose is …digital literacy or whatever… Some people might have difficulty doing that and therefore not engage if they find that they're having to download another app on their phone or access something and they're not sure if they've submitted this or you know, certain people will have be less comfortable dealing with those sorts of things.” S7 |
|  |  | “I just think some older Australians aren’t going to be that tech savvy in Australia.” P5 |
|  | Staff perceived additional barriers for patient engagement with the DCP, including low health literacy, survey fatigue, low motivation, lack of human contact and lack of follow up for completers. | “it might be a bit annoying for them …they've already talked to the clinician or the nurse or the video or whatever, about their back pain and then they get a text message to ask the same questions, that might be a bit challenging. …The only concern is that when it's an automated text message that you can't reply to or it's not a person on the other end, I think that sometimes gets a bit old and easy to ignore… and I can easily block that number and I have, so you can easily ignore it. If it's not perceived as something that cares about you individually, I suppose. …I think people find it very challenging to see what the point of them participating is when hopefully for them [it] won't influence them again with their back pain. …I suppose if you're still suffering with back pain at three months, and the hospital is not really supporting you anymore. For whatever reason, if you're …not going to the back pain clinic or anything anymore just for acuity. Then you might kind of feel a bit that you've been a bit neglected I suppose. They're still giving the hospital data but they haven't had their back pain fixed which we know is inevitable for some of the patients.” S4 |
|  |  | “I think for a lot of people, the pain goes away. And then they forget about it, then once they're in that mindset, they might not do a follow up questionnaire” S3 |
|  |  | “Using digital removes the human side a little bit further and they might feel less listened or appreciated.” S7 |
|  |  | “Sometimes people come to the appointment and they don't really know even what it's for. …’where's my GP in all of this or even do I have a GP?’ So a bit of confusion there... And their presentation from ED might not be or frequently would not be their first contact with the health system about this problem... health literacy [is an issue] but sometimes just literacy even in their own language” S6 |
|  |  | “I attribute a lot of the difficulties that people experience just to health literacy. Alone and misunderstanding the emergency department’s ability to help them with their complaints because their health literacy is terrible. I just think we need to recognize that people come into ED because to them, they've got a significant complaint. That may not be life threatening, but to them, it feels it feels very threatening. I just think the messaging needs to recognize the stage of understanding that people are up to at that point … The biggest and most obvious [challenge] is just language and health literacy. Engagement. Perceived, I guess the benefit of ‘what am I trying to get out of this?’” S2 |
|  |  | “And for the more discerning ones, I think they will question why they're still being contacted when they're not having treatment, but I think that will be a small percentage.” S1 |
|  | Two staff members perceived that the introduction of the DCP may lead to additional administrative burden. One of the two staff members were also concerned about additional clinical burden. | “from the admin staff point of view I think they already collect [people’s phone numbers] …as a data point. I think if we made an email that might be slightly more challenging because I don't think we have email in general and you know, you have to spell names and that's there's very there's a lot of transcription errors that can happen with email. I think from a staff point of view, I can't imagine that it would make a big influence on what we do, …I would hope that any staff member that is seeing a patient with back pain is already asking those questions. S4 |
|  |  | “additional time required by the clinician to interpret those results and be able to apply them to improve the way that they provide care. And in this clinic, [we] don't really have admin [support]. So any additional administrative tasks will fall on the clinician as well. So yeah, there the two kind of challenges I see. …time required other than seeing the patients, doing their notes and the follow up to interpret the problems and then to any other additional admin that comes with that would fall on the clinician.” S1 |
| Facilitators and recommendations for improving engagement with the DCP. | Participants report that the main motivation for patients to engage with DCP surveys was altruism. However, one staff member had contrasting views. Patients also report tracking progress and reducing the risk of reoccurrence as other facilitators. | “I've been obviously doing [exercises] everyday because I don't want that pain …for me the [survey] score wouldn't really worry me, just knowing that …people have seen it and like ‘oh, okay, well, this has helped’. That's just more motivational for me because it's like I've helped somebody with that. …just having that extra knowledge, being able to give my opinion and how I've tracked doing things that I've been shown or that has been suggested to me like that having that help others would …reduce anxiety over the pain that you're having” P3 |
|  |  | “I like doing these things. You know, helping the community... Bigger picture, hopefully everybody's doing it so you guys can have a good database. So that will be my motivation.” P2 |
|  |  | “I guess I just want to try and help the next person that might have back pain so they know what they're going through, it's probably just more helping someone else... Because that's what makes me do all my exercises and things because I'm like, I just sit there and go ‘Did you want that pain again? No, get up and do your exercises.’... That way, I can also look back and see it, too, whereas at the moment, it's in my head, that I know it's getting better but if it's all collected by you, and I can see it, I'm like, Oh, look at that [graph]” P4 |
|  |  | “If I feel that it's going to benefit me and it's not just, you know, some sort of like a survey thing that I'm doing for research, I guess so like, you know, you go and shop some and they say, can you do this feedback? I mean, I definitely will do it once because I want to help the program and so on” P6 |
|  |  | “I think people in general are motivated to help out others. And so I think as long as it's in a usable format, and people understand how the data why the data is being collected. I think I would hope that it wouldn't be too much of an imposition of them.” S4 |
|  |  | “I'm not sure if participating in research is a good enough driver or strong enough driver.” S5 |
|  | Participants felt that resources such as videos and personal results would increase patient engagement with the DCP | “I think videos are a good move, and short is in the right direction” S1 |
|  |  | “[a reassurance video] sounds fantastic.” S2 |
|  |  | “as long as I fill out the questions that are related to the injury, and the recovery process for my injury and perhaps exercises... that's all relevant. That's good. I’d find that engaging... graphs and data and statistics and all that on my improvement...It's also a good reference point” P7 |
|  |  | “I just think that angle I would try to take from is it's about the patients. We're trying to help them. We're trying to give them resources to help get them back up and running. And as a part of that, we do need them to do some data, enter a few stats, reply to a text message every now and then. Perhaps get people over the line if they're reluctant otherwise” S2 |
|  |  | “So I think in terms of the expectation video, personally, I think that … that's very important in back pain as I've already mentioned.” S4 |
|  | Continuity of care through periodic contact with health professionals was important and perceived to potentially improve engagement with the DCP. | “So you know what it is, you can touch it, as you know, there will be someone calling you to monitor this and what it does is that it gives them I think the value add is that they actually have input from specialists and constant contact. So if you can work it into the survey, or perhaps another way of engagement is that someone calls them and ask them the questions. But I don't know if it's your digital purpose. It's just I think that engagement during [the DCP], someone's calling who cares, who knows what they're talking about, and offer them good advice about how to get better? I feel that will be a great motivator.” S5 |
|  |  | “…that's kind of an ongoing benefit that you're getting that feeling that there is still somebody out there that cares that you're suffering, and so that kind of feeling of nurture, I suppose.” S4 |
|  |  | “[If] I didn't understand something, being able to call someone, say ‘look, on this page, it had this I'm not quite sure what you mean.’ And [have that] explained to me, I'm like, oh, okay, and then I can give a better more in depth answer to that question.” P3 |
|  |  | “You know, like I said, even you know after a week or so, and then that's it stop until comes again, but who do who do we contact, …what to do when we have the pain, you know?” P8 |
|  |  | Maybe like to continue the journey because I mean, without that, how can they provide further support? If this escalates with me trying to manage this digitally, and it's just not going anywhere? And I go to a doctor and they don't have any of this data, then they will probably have to start the X rays or the scans and all that kind of stuff. And even if they do that, they will have information in front of them the progression of where I started, where I am now, to make the call in what next? P6 |
|  |  | “I guess if you really dig deep into it from a psychological perspective, it's nice to have that one-on-one experience every now and again. Just to check in to see am I doing the right thing and ask questions and just that support.” P6 |
|  |  | “No, if it's adding to this we have a video call. Maybe Yeah, good. It's because I think back pain they have to see or touch or massage to to know exactly where the pain is.” P2 |
|  | Staff reported having an empathetic approach to managing patient expectations may improve engagement. One participant pointed out that patients would respond positively to short waiting times for clinic appointments. | “I think if from the start, you can encourage patients that this is a conversation about trying to empower them to get them home. I think if you said that from the start with a shared kind of mental approach to their suffering for whatever reason, you're better down the track than if you just jump into the ‘how bad [is] your pain, what's happening’ because then when you get to the end, ‘it's okay you can go home’ and people aren't expecting that, they think their condition warrants being in hospital even though we know that there's not really a lot we do in hospital necessarily. …Yep, helping other people, I suppose reflecting themselves on their back pain. I always say this to patients that they say, ‘I've been suffering for this long’ or however long, and there's no actual record of that, obviously, because it's just been a kind of gut feeling. I suppose with this specific time point, you know, if at six weeks’ time that like I'm feeling better, that would hopefully reflect to them as well, like I hope for if it happens again, then you're going to it's going to improve in terms of incentivizing them.” S4 |
|  |  | “…I guess the thing that's tricky is it's nuanced for every patient who comes in …blanket reassurance isn't nuanced enough to capture the doubt that people have, that you've misinterpreted their symptoms. And you've misinterpreted their underlying complaint.” S2 |
|  |  | “What I think might be helpful psychologically, perhaps is to tell them how long it will take actually, that sort of gives them an idea of how much time they're spending on this...” S5 |
|  |  | “I think if I was to sort of go with what I would share with the patients, let's say it would be hopefully modifying expectations is a huge issue because I think a lot of the back patients that I see have unrealistic expectations that we're going to fix them. And the first thing we have to do is that we'll try and say we're going to manage this issue or we're going to try and help improve but you know, we're not going to wave a magic wand and fix this today. So there's often getting people to modify their expectations.” S7 |
|  |  | “At this point, they don't know what a physio [is], that I'm a specialist in back pain. We will see you in two weeks. And they're rapt by that because normally they get told, …you can see the specialist and that will be in three months’ time. So it gets real buy in from these patients.” S1 |
